# Green Chemistry Production of Codlemone, the Sex Pheromone of the Codling Moth (*Cydia pomonella*)*,* by Metabolic Engineering of the Oilseed Crop Camelina (*Camelina sativa*)

**DOI:** 10.1007/s10886-021-01316-4

**Published:** 2021-11-11

**Authors:** Yi-Han Xia, Hong-Lei Wang, Bao-Jian Ding, Glenn P. Svensson, Carin Jarl-Sunesson, Edgar B. Cahoon, Per Hofvander, Christer Löfstedt

**Affiliations:** 1 Department of Biology, Lund University, Sölvegatan 37, 22362 Lund, Sweden; 2 Department of Biochemistry and Center for Plant Science Innovation, University of Nebraska-Lincoln, Lincoln, NE 68588 USA; 3 Department of Plant Breeding, Swedish University of Agricultural Sciences, P.O. Box 101, 23053 Alnarp, Sweden; 4 Present Address: Division of Systems and Synthetic Biology, Department of Biology and Biological Engineering, Chalmers University of Technology, 41296 Gothenburg, Sweden

**Keywords:** Conjugated double bonds, Plant factory, Acyl-ACP thioesterase, ∆9 desaturase, Multi-gene copies, P19, *Agrobacterium*-based floral-dip transformation, Bioassay

## Abstract

Synthetic pheromones have been used for pest control over several decades. The conventional synthesis of di-unsaturated pheromone compounds is usually complex and costly. Camelina (*Camelina sativa*) has emerged as an ideal, non-food biotech oilseed platform for production of oils with modified fatty acid compositions. We used Camelina as a plant factory to produce mono- and di-unsaturated C_12_ chain length moth sex pheromone precursors, (*E*)-9-dodecenoic acid and (*E*,*E*)-8,10-dodecadienoic acid, by introducing a fatty acyl-ACP thioesterase FatB gene *UcTE* from California bay laurel (*Umbellularia californica*) and a bifunctional ∆9 desaturase gene *Cpo_CPRQ* from the codling moth, *Cydia pomonella*. Different transgene combinations were investigated for increasing pheromone precursor yield. The most productive Camelina line was engineered with a vector that contained one copy of *UcTE* and the viral suppressor protein encoding *P19* transgenes and three copies of *Cpo_CPRQ* transgene. The T_2_ generation of this line produced 9.4% of (*E*)-9-dodecenoic acid and 5.5% of (*E*,*E*)-8,10-dodecadienoic acid of the total fatty acids, and seeds were selected to advance top-performing lines to homozygosity. In the T_4_ generation, production levels of (*E*)-9-dodecenoic acid and (*E*,*E*)-8,10-dodecadienoic acid remained stable. The diene acid together with other seed fatty acids were converted into corresponding alcohols, and the bioactivity of the plant-derived codlemone was confirmed by GC-EAD and a flight tunnel assay. Trapping in orchards and home gardens confirmed significant and specific attraction of *C. pomonella* males to the plant-derived codlemone.

## Introduction

Pheromones have been used in Integrated Pest Management (IPM) for several decades (Trematerra 1997; Witzgall et al. 2010) as environmentally friendly, species-specific, non-toxic, and with a low risk of pests evolving resistance. The global agricultural pheromones market size was valued at $ 1.9 billion in 2017 and is estimated to reach $ 6.2 billion by the end of 2025 (www.fortunebusinessinsights.com). Conventional industrial synthesis of pheromones may result in hazardous byproducts and residues (Mori 2007, 2010). A green chemistry alternative for pheromone production has become increasingly attractive and feasible during the last two decades. Multiple examples of production of C_16_ and C_14_ moth pheromone compounds have been reported. These include: (1) production of pheromone precursors in *Nicotiana* spp. (Nešněrová et al. 2004; Xia et al. 2020), (2) production of biologically active multi-component moth sex pheromones in *N. benthamiana* (Ding et al. 2014), (3) production of moth pheromones by fermentation in *Saccharomyces cerevisiae* (Hagström et al. 2013) and *Yarrowia lipolytica* (Holkenbrink et al. 2020), and (4) production of an aphid alarm pheromone (*E*)-β-farnesene in Arabidopsis (Beale et al. 2006) and its release from wheat under field conditions (Bruce et al. 2015).

Most known moth pheromones are of the so-called “Type I”, fatty acid derivatives that have C_10_ to C_18_ carbon chain-lengths with up to three double bonds and an oxygenated functional group (Ando et al. 2004; Löfstedt et al. 2016). To date, more than 500 species of moths have been found to use C_12_ chain-length pheromones in their chemical communication systems (Ando 2021), of which many are serious pests on high value crops, including the codling moth (*Cydia pomonella*), the oriental fruit moth (*Grapholita molesta*), and the European grapevine moth (*Lobesia botrana*). Here, we explore the use of the oilseed crop Camelina (*Camelina sativa*) as a production platform for C_12_ fatty acid pheromone precursors with a focus on the doubly unsaturated sex pheromone component of the codling moth.

Some of the C_12_ moth pheromones are biosynthesized in insects by introduction of double bonds in longer fatty acid homologues followed by subsequent chain shortening (Bjostad and Roelofs 1983) and modification of the functional group, whereas others are biosynthesized by direct desaturation of lauric acid (12:0) and conversion of the immediate fatty acyl precursor into the actual pheromone component(s). The latter alternative holds for codlemone, the major pheromone component of the codling moth, *C. pomonella,* identified as (*E*,*E*)-8,10-dodecadienol (E8,E10-12:OH) by Roelofs et al. (1971). A ∆9 desaturase gene *Cpo_CPRQ* acting on 12:0 is pivotal in codlemone biosynthesis, accounting for both double bonds in even positions (Löfstedt and Bengtsson 1988; Lassance et al. 2021). To optimize seeds of oil crops for the production of medium-chain fatty acids (MCFA, C_6_–C_12_ fatty acids), fatty acid biosynthesis needs to be redesigned to generate maximal levels of 12:0, rather than the typical C_16_ and C_18_ fatty acids found in triacylglycerols (TAG) of these seeds. In plants, the biosynthesis of MCFA is a variation on typical de novo fatty acid synthesis that generates primarily C_16_ and C_18_ fatty acids. Chain-lengths of fatty acids released from the plastids in plants are primarily determined by acyl-ACP thioesterases, including FatB thioesterases that typically release C_16_ acyl chains from de novo fatty acid biosynthesis (Li-Beisson et al. 2013). Variant forms of FatB are able to release fatty acids of chain lengths shorter than C_16_ (Jones et al. 1995; Kim et al. 2015a; Pollard et al. 1991; Tjellström et al. 2013; Voelker 1996). In a previous study, a FatB gene *UcTE* from California bay laurel (*Umbellularia californica*) was found to have high activity for production of 12:0 in rapeseed (*Brassica napus*) (Voelker et al. 1992).

We engineered the pathways towards the production of the immediate fatty acyl precursor of the codling moth pheromone, (*E*8,*E*10)-dodecadienoic acid (E8,E10-12:acid), in Camelina seeds. Camelina was chosen as the oilseed production platform for our studies because it is of limited use as a food crop and is considered an ideal system for rapid introduction and evaluation of fatty acid and other oil-related traits (Iskandarov et al. 2014). Foremost, transgenes can easily be introduced into Camelina using a simple *Agrobacterium*-based method (Lu and Kang 2008), and it has a relatively short life cycle that allows up to three generations in a year for evaluation of engineered traits (Bansal and Durrett 2016). Camelina is also closely related to *Arabidopsis thaliana*, with a wealth of transgenic and genomic data for optimizing endogenous biosynthetic pathways for production of desired oil traits in seeds that typically are 30% to 40% oil by weight (Nguyen et al. 2013). To explore oilseed production of a high amount of C_12_ pheromone precursors, we co-expressed the *UcTE* and *Cpo_CPRQ* in Camelina seeds for production of (*E*)-9-dodecenoic acid (E9-12:acid) and E8,E10-12:acid using four different strategies. The biological activity of plant-derived codlemone so obtained was demonstrated by electrophysiological and field trapping experiments, supporting the feasibility of producing the codlemone precursor and other C_12_ pheromone precursors in stably transformed plant seeds.

## Materials and Methods

### Plant Material and Growth Conditions

For these studies, *C. sativa* cv. Suneson (Camelina) was used. The previously described high lauric (20 mol% of total seed fatty acids) Camelina line was used as our primary metabolic engineering platform (Kim et al. 2015a). Two plants per pot (20 cm diameter, 20 cm deep) with soil were grown under greenhouse conditions of approximately 24 °C, 16 h day/18 °C, 8 h night, with supplemental lighting (400–500 μmoles/m^2^/s) as needed to maintain day length conditions.

### Insects and Insect Extracts

The pupae of codling moths were purchased from Andermatt Biocontrol AG (Switzerland). After being sexed, the male and female pupae were kept separately and emerged at 23 ± 1 °C, 17:7 L/D and 70% relative humidity. 2- to 3-day-old adults were used in the experiments. For pheromone extraction, 5–6 pheromone glands of virgin females were dissected 1–2 h into the scotophase, and extracted in 50 µL of n-heptane for 30 min.

### Gene Cloning—Preparation of Constructs

The open reading frames (ORFs) of *UcTE* (Genebank accession number: Q41635.1) and codon optimized-*CpoCPRQ* were synthesized by Invitrogen. The sequence of three seed-specific promoters for the α’-subunit of β-conglycinin gene (*β-con*) (Chamberland et al. 1992), β-phaseolin gene (*ß-Phaseolin*) (van der Geest and Hall 1996), oleosin gene (*Oleosin*) (Fan et al. 2013), and two terminators for nopaline synthase gene (*NOS*), and nopaline synthase gene (*HSP*) were also synthesized by Invitrogen. ORFs of *CpoCPRQ* (AHW98354), *AtWRINKLED1* (AY254038), *CvLPAAT* (ALM22867), *P19* (P69516.1), and sequence of napin gene promoter (*Napin*), octopine synthase terminator (*OCS*) were amplified from entry clones. The glycinin gene promoter (*Glycinin*) was already contained in the final expression vector pBinGlyBar (Nguyen et al. 2013).

**Fig. 1:**
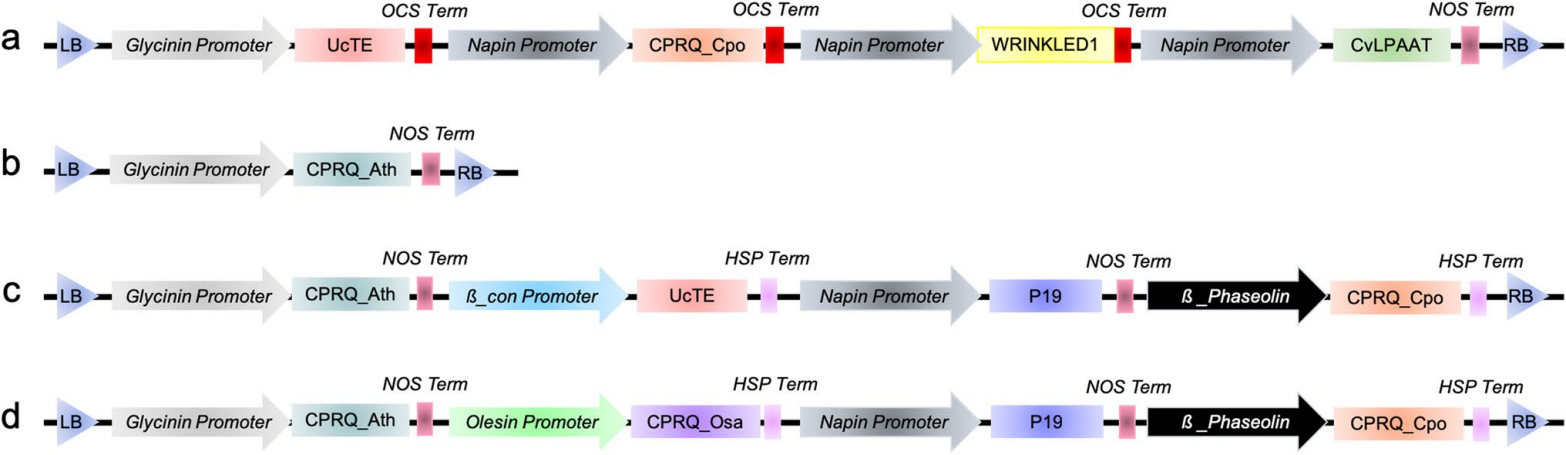
Scheme of gene cassettes from four engineering strategies towards pheromone precursor production in *C. sativa*. Gene cassette of a CPRQ1.0; b CPRQ1.1; c CPRQ2.1; d CPRQ2.2 Glycinin Promoter, *Glycine max* glycinin gene promoter; Napin Promoter, *Brassica napus* napin gene promoter; ß_con Promoter, *Glycine max* ß_conglycinin gene promoter; ß_Phaseolin Promoter, *Phaseolus vulgaris* ß_Phaseolin gene promoter; Oleosin Promoter, *Brassica napus* oleosin gene promoter; OCS Term, *Agrobacterium* octopine synthase terminator; NOS Term, *Agrobacterium* nopaline synthase terminator; HSP Term, *A. thaliana* heat shock protein terminator. *Cpo_CPRQ_Ath* and *Cpo_CPRQ_Osa* means that *Cpo_CPRQ* is codon optimized for *A. thaliana* and *O. sativa*, respectively

To compare different strategies towards production of high amounts of C_12_ pheromone precursors in Camelina, four plant expression vectors were constructed (Fig. 1). (1) CPRQ1.0 contained four exogenous genes of which *UcTE* was controlled by *Glycinin* and *Cpo_CPRQ*, *AtWRINKLED1* and *CvLPAAT* were controlled by *napin* promoter (Fig. 1a). (2) CPRQ1.1 contained one exogenous gene *Cpo_CPRQ* codon optimized for *A. thaliana* (Arabidopsis) (*Cpo_CPRQ_Ath*) and controlled by *Glycinin* (Fig. 1b). (3) CPRQ2.1 contained four exogenous genes of which *Cpo_CPRQ_Ath* was controlled by *Glycinin*, *UcTE* was controlled by *β-con*, P19 was controlled by *Napin* and *Cpo_CPRQ* was controlled by *ß-Phaseolin* (Fig. 1c). (4) CPRQ2.2 also contained four exogenous genes, which were three copies of *Cpo_CPRQ* (one without codon optimized, one codon optimized for Arabidopsis, one codon optimized for *Oryza sativa*) controlled by *ß-Phaseolin*, *Glycinin*, *Oleosin*, respectively, and a *P19* controlled by *Napin* (Fig. 1d). CPRQ1.0 was transformed into wild type Camelina, whereas the other three vectors were transformed into high lauric acid type Camelina.

PCR amplification was performed using the entry clone as template with a pair of degenerate primers, on a Veriti Thermo Cycler, using Phusion Flash High-Fidelity PCR Master Mix (Thermo Scientific™) under conditions as follows: start at 98 °C for 30 s, and 38 cycles at 98 °C for 5 s, 55 °C for 10 s and 72 °C for 50 s, followed by a final extension step at 72 °C for 10 min. Subsequently, fusion PCR was performed using phusion®Taq (Thermo Scientific™) (Atanassov et al. 2009) to do truncation and gene fusion for gene assembly, using the same PCR programs as described before. All genes with promoters and terminators were cloned into the plant expression vector pBinGlyBar, which contained a *bar* marker gene for Basta selection of transformed plants, by using Multisite Gateway® recombination cloning technology (Invitrogen). The constructed expression clones were confirmed by sequencing.

### Floral Transformation of Camelina by Agrobacterium

The constructed expression vectors were introduced into *Agrobacterium tumefaciens* strain GV3101 (MP90RK) by electroporation (1700 V mm^−1^, 5 ms, Eppendorf 2510). The transformed *Agrobacterium* cells were grown on solid LB medium supplemented with antibiotics (50 mg/L rifampicin, 50 mg/L gentamicin and 50 mg/L spectinomycin) after incubating at 30 °C for 36 h. Afterwards, a single clone from each expression clone was incubated in 2 mL liquid LB medium with antibiotics as described above at 30 °C for 36 h. The *Agrobacterium* solution was then transferred to 30 mL medium for a 36 h incubation, and after that, the solution was transferred to 1 L medium for a 24 h incubation. Subsequently, the 5 weeks old Camelina plants were transformed by the floral vacuum infiltration method as described by Lu and Kang (2008) and Liu et al. (2012). A Basta resistance gene was used as a selection marker (Nguyen et al. 2013).

### Sampling for Seed Oils Analysis

Seeds harvested from the floral-dipped plants were sown in soil for Basta selection, and the surviving T_1_ plants were considered as transformants. T_2_ seeds were harvested from matured T_1_ plants. Twenty-five T_2_ seeds from each T_1_ plant were randomly selected for pooled seeds fatty acid analysis. After the analysis, the four most productive plants from each strategy were identified. Fifteen individual seeds from the identified plants were randomly selected for single seed fatty acid analysis. Then, four plants from the best strategy line were selected for further propagation. For each plant, 25 seeds were sown for producing T_3_ seeds. Similarly, 25 T_3_ seeds from each T_2_ plant were randomly selected for pooled seeds fatty acid analysis, and 15 individual seeds from the three most productive plants were randomly selected for single seed fatty acid analysis. Afterwards, 20 to 45 seeds from the three T_3_ plants were sown to produce T_4_ seeds. Likewise, 25 T_4_ seeds from each T_3_ plant were randomly selected for pooled seeds fatty acid analysis. As the T_4_ seeds upon analysis could be considered homozygous, the seeds from all mother plants (T_3_ plants) were also pooled for fatty acid analysis and subsequently used for isolation of fatty acids for biotests.

### Fatty Acid Analysis of Seed Oils

Fatty acids were analyzed as fatty acid methyl esters (FAMEs), which were generated from putative transformants either by grinding 25 pooled seeds (for production analysis of one transformant) or by grinding 15 seeds individually (for variation analysis within one transformant) from each mother plants in 1 mL 2% H_2_SO_4_ in methanol in a 4 mL glass vial. After grinding, the samples were incubated for 1 h at 90 °C. After cooling down to room temperature, 1 mL water and 1 mL heptane were added and the vial was vortexed. Then, the heptane phase containing the FAMEs was transferred to a new Agilent vial for GC/MS analysis on an Agilent 5975 mass-selective detector, coupled to an Agilent 6890 series gas chromatograph equipped with a polar column (HP-INNOWax, 30 m × 0.25 mm, 0.25 μm), and helium was used as carrier gas. The oven temperature was set at 80 °C for 1 min, then increased to 230 °C at a rate of 10 °C/min and held for 10 min. Fatty acid compounds were identified by comparison of retention times and mass spectra with those of reference compounds. To determine the position of double bonds in unsaturated fatty acids, DMDS derivatization was performed according to Dunkelblum et al. (1985). The DMDS-adducts were analyzed by GC/MS on a non-polar column (HP-5MS, 30 m × 0.25 mm, 0.25 μm) under the following oven temperature program: 80 °C for 2 min, then increased at a rate of 15 °C/min to 140 °C, and then increased at a rate of 5 °C/min to 260 °C, and held for 30 min.

### Isolation and Reduction of Acids for Biotests

T_4_ seeds from 44 plants of line CPRQ2.2_5-3, 48 plants of line CPRQ2.2_5-12 and 18 plants of line CPRQ2.2_9-26 were harvested. After acid methanolysis similar to that described above, the methanolysis product in heptane was additionally washed with 1 mL of water, and then dried over anhydrous sodium sulfate before GC/MS analysis.

FAME samples were converted into alcohols in different batches. Batch 1 was a pooled FAME sample from 44 plants of line CPRQ2.2_5-3 and 48 plants of line CPRQ2.2_5-12, combined in a total volume of ca. 20 mL, which contained ca. 3% of the E8,E10-12:Me. After solvent evaporation, approximately 140 mg of FAMEs were obtained. To reduce fatty acid methyl esters to the corresponding alcohols, 3.4 mL anhydrous diethyl ether was added in a 50 mL flask under a nitrogen atmosphere and cooled with an ice-salt bath to 0 °C, while lithium aluminum hydride (39.3 mg, 1.04 mmol, ~ 2.0 equiv) was added. After stirring for 10 min, the 140 mg FAME sample dissolved in 1 mL of anhydrous diethyl ether was added into the flask. The mixture was stirred for 15 min and then warmed to room temperature and continued stirring for 2 h. After reaction, the reduction mixture was cooled with ice bath, and treated by successive addition of 10 µL of water, 10 µL of 15% sodium hydroxide solution and 30 µL of water, followed by stirring for 15 min at room temperature. The crude product was dried over anhydrous sodium sulfate and purified by flash chromatography on silica gel 60 (70–230 mesh) before analysis by GC/MS. This batch of plant-derived alcohol product was used in the electrophysiological experiment (see below).

The second batch of FAME samples, extracted from 5.1 g seeds from 48 plants of line CPRQ2.2_5-12, was directly reduced and the crude alcohol product was used in the first field trapping experiment without further purification.

The third batch of FAME samples, from 1.05 g seeds of line CPRQ2.2_5-12, was first fractionated by silver nitrate-silica column (15 cm, 2 mm i.d., 10% AgNO_3_) before reduction. The column was eluted consecutively with 4 mL mixture of heptane/acetone (98:2) as mobile phase, followed by another 4 mL heptane/acetone (96:4). The fractions that contained the target compound E8,E10-12:Me as the major component (above 85%) were combined and reduced to alcohol as described above.

### Electrophysiology

The antennal electrophysiological responses of male *C. pomonella* to the plant-derived codlemone, to gland extracts of female adults, as well as synthetic codlemone were recorded on an Agilent 7890 gas chromatograph equipped with a flame ionization detector (FID) (Agilent, Santa Clara, California) and an electroantennographic detector (EAD). An HP-INNOWax column (30 m × 0.25 mm, 0.25 μm) was used in the GC. Hydrogen was used as the carrier gas with a constant flow of 1.8 mL/min, and a 1:1 division of the GC effluent was directed to the FID and EAD. The inlet was set at 250 °C, the transfer line was set at 255 °C and the detector was set at 280 °C. A PRG-2 EAG (10 × gain) probe (Syntech, Kirchzarten, Germany) was used in the recordings. Both tips-cut antennae from a male adult, associated with the head were mounted to the probe using conductive gel (Blågel, Cefar, Malmö, Sweden), and the antennal preparation was put in a flow of charcoal-filtered and humidified air (velocity ca. 25 cm/s). The GC oven was programmed from 80 °C for 1 min, then increased to 210 °C at a rate of 10 °C/min and held for 10 min. Data were collected with the software GC-EAD Pro Version 4.1 (Syntech).

### Field Trapping

A first round of field trapping experiments was carried out in two apple orchards in southern Sweden, a small orchard (ca. 2 ha, 55° 33.55 N, 14° 18.67 E) and a bigger orchard (> 10 ha, 55° 29.70 N, 14° 17.24 E), and in home gardens in Lund municipality (55° 42.31 N, 13° 11.48 E) from June 9th to 17th, 2021. Delta-traps with sticky inserts (Csalomon, Budapest, Hungary) and red rubber septum (Catalogue no. 224100-020, Wheaton Science Products, Millville, NJ, USA) dispensers were used. Plant-derived codlemone and synthetic codlemone (sample of unknown origin from our laboratory collection of reference compounds; isomeric purity 97.7% EE, 0.4% EZ, 1.3% ZE and 0.6% ZZ) were loaded on rubber septa at a dose of 100 µg/septum of the E8,E10-12:OH (AI), whereas 100 µL of n-heptane alone was loaded as a negative control.

A second round of field trapping was carried out in home gardens in Lund municipality (as specified above) from June 24th to 28th, 2021 to compare the activity of plant-derived codlemone after additional purification (mainly removal of C_18_ alcohols and improvement of isomeric purity as described above) with the initially obtained plant-derived codlemone. Conventionally produced codlemone (same as in previous experiment) was used as a positive control.

Traps within a replicate were randomly suspended in a row of apple trees in the orchards, at a height of ≈ 2 m above ground, with a distance of 10–12 m between traps and 12 m between rows. For experiments in home gardens, one trap with each treatment (or control) was hung in one tree at ≈ 2 m above ground and separated by at least 2 m. Distance between home gardens (and thus between replicates) was ≥ 100 m. The trapped moths were identified by their morphological characters. Catch data were log(x + 1)-transformed before performing statistical analysis. In the first experiment, catch data were compared using t-test, whereas catch data in the second experiment were compared using Anova followed by the Bonferroni *post-hoc* test (SPSS ver. 27).

### Flight Tunnel Assay

The behavioral response of male *C. pomonella* to the plant-derived and synthetic pheromone was observed in a Plexiglas flight tunnel size 0.9 × 0.9 × 3 m (Valeur and Löfstedt 1996). The experimental conditions were: temperature 20 ± 1 °C, relative humidity 30 ± 2%, wind speed 0.3 m/s and light intensity 3.6 lx. A rubber septum was used as dispenser on which 100 µg of plant-derived crude or purified codlemone, or synthetic codlemone dissolved in 100 µL n-heptane were loaded (similar to baits used for field experiments). Experiments were conducted 0–1.5 h into the scotophase using 2–3 days old virgin males. A male was introduced in the downwind end of the flight tunnel and held in the pheromone plume for approximately 5–10 s, and allowed to take off and then observed for 3 min. For each treatment group, 15–20 males were tested, and each male was used only once. Differences in proportion of males reaching the odor source were analysed by the Chi-square test (SPSS ver. 27).

## Results

### Assembly of Pathways for Moth Pheromone Precursor Biosynthesis and Accumulation in Camelina Seeds

**Fig. 2:**
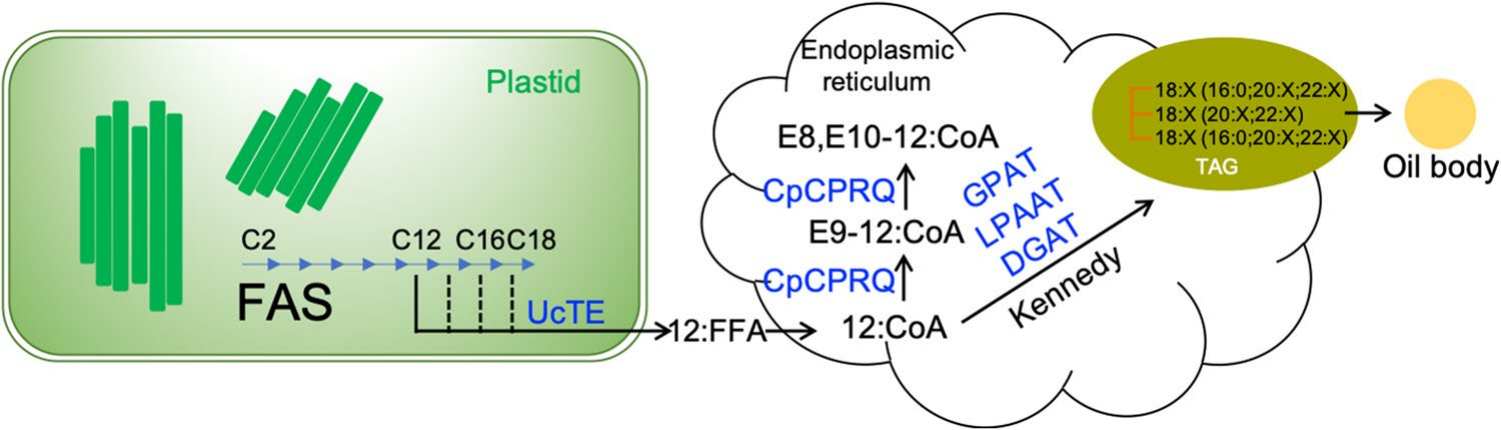
Engineered pathways for production of mono- and di-unsaturated C_12_ pheromone precursors in Camelina seeds. FFA, free fatty acids. TAG, triacylglycerol. The introduced enzymes are indicated in blue. Acyl intermediates in the pathway are given as short forms. E9 -12:CoA refers to the fatty-acyl coenzyme A with a chain length of 12-carbon atoms and a double bond at ∆9 position in *E* configuration; E8,E10-12:CoA refers to the fatty-acyl coenzyme A with a chain length of 12-carbon atoms and two conjugated double bonds at ∆8 and ∆10 positions in *E* configuration

The engineered pathway for pheromone precursor production is shown in Fig. 2. The thioesterase encoded by *UcTE* releases lauric acid (12:0) from plastid acyl carrier protein (ACP), effectively stopping further fatty acid chain elongation. The released 12:0 is activated with coenzyme A upon transport from the plastid to cytosol, and the resulting 12:0-CoA serves as the substrate for production of E9-12:CoA, and E8,E10-12:CoA by the desaturase encoded by *CpCPRQ*. We investigated four strategies towards the production of these two compounds in the plant factory: (1) co-expression of *CpCPRQ* with a seed-specific synthetic transcription factor Wrinkled1 (Wri1) *A. thaliana* enhancing fatty acid synthesis and a Kennedy pathway gene, lysophosphatidic acid acyltransferase gene *CvLPAAT* from *Cuphea viscosissima (*Kim et al. 2015b); (2) transformation of only one gene copy of *CpCPRQ* into high lauric Camelina seeds; (3) transformation of multiple gene copies with different promoters and terminators; and (4) stable expression of the viral gene silencing suppressor protein P19 together with pheromone biosynthetic genes.

### C_12_ Pheromone Precursors were Produced in Camelina Transformants

**Fig. 3:**
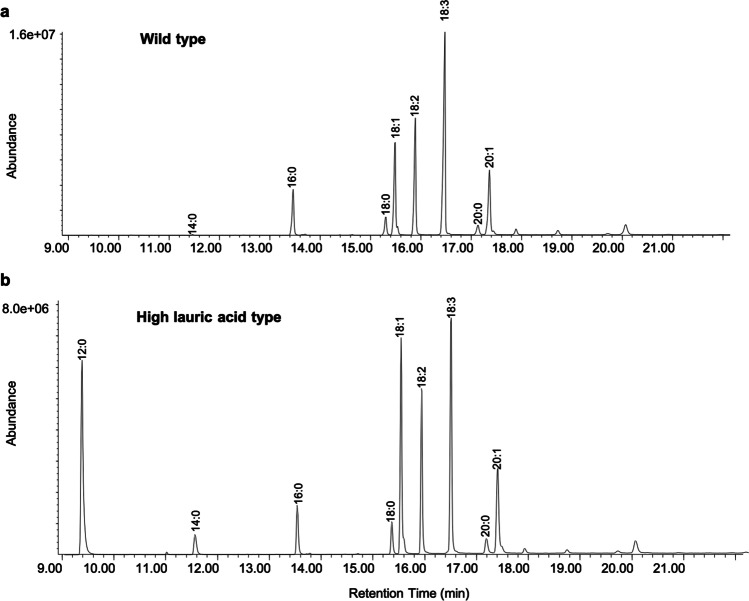
Chromatograms of fatty acid profiles in Camelina seeds from **a** wild type and **b** high lauric acid type. GC/MS analysis of total fatty acids in the form of corresponding methyl esters. 14:0. myristic acid; 16:0, palmitic acid; 18:0, stearic acid; 18:1, oleic acid; 18:2, linoleic acid; 18:3, linolenic acid; 20:0, arachidic acid; 20:1, gondoic acid

GC/MS analysis of C_12_ to C_20_ chain length fatty acids showed that in wild type Camelina seeds, linolenic acid (18:3) was the most abundant fatty acid, followed by linoleic acid (18:2) and oleic acid (18:1) (Fig. 3a and Table 1). The second most abundant group of fatty acids comprised gondoic acid (20:1), palmitic acid (16:0) and stearic acid (18:0). In addition, small amounts of lauric acid, myristic acid, and arachidic acid were found in the seeds (Fig. 3a and Table 1). In the high lauric acid (12:0) Camelina seeds, the amount of 12:0 was as high as 18:3, and 18:1 was the second most abundant fatty acid (Fig. 3b and Table 1).

**Table 1:** Mole percentage range of C_12_ to C_18_ fatty acid composition in seed lipids in T_2_ transformants, wild type and high lauric acid type

Genotype	N value^a^	12:0	E9-12	14:0	E8,E10-12	16:0	18:0	18:1	18:2	18:3
Wild type	5	0	–	0.1–0.2	–	7.0–10.5	2.8–4.8	13.9–18.8	15.8–20.7	32.6–42.7
High lauric acid type	5	22.9–31.4	–	2.9–4.2	–	4.0–7.7	2.1–3.9	13.8–19.8	10.1–15.2	16.5–22.7
CPRQ1.0	9	3.4–11.1	0.1–2.5	0.13–2.0	0.01–0.22	6.1–8.6	1.4–3.1	8.2–19.4	17.5–29.4	23.9–37.7
CPRQ1.1	85	10.1–29.7	0.2–2.4	0.9–4.7	0.01–0.22	6.1–12.2	1.6–4.0	6.0–18.2	11.3–22.4	19.3–31.6
CPRQ2.1	95	25.4–38.4	0.2–3.4	2.5–4.0	0.01–0.31	4.6–6.3	1.2–2.6	7.6–12.8	11.1–14.4	18.8–27.1
CPRQ2.2	30	19.8–38.8	1.0–10.6	2.0–6.4	0.13–5.50	4.4–7.4	1.1–1.7	8.6–16.8	10.3–15.9	19.2–29.3

^a^Each sample containing 25 pooled seeds

**Fig. 4:**
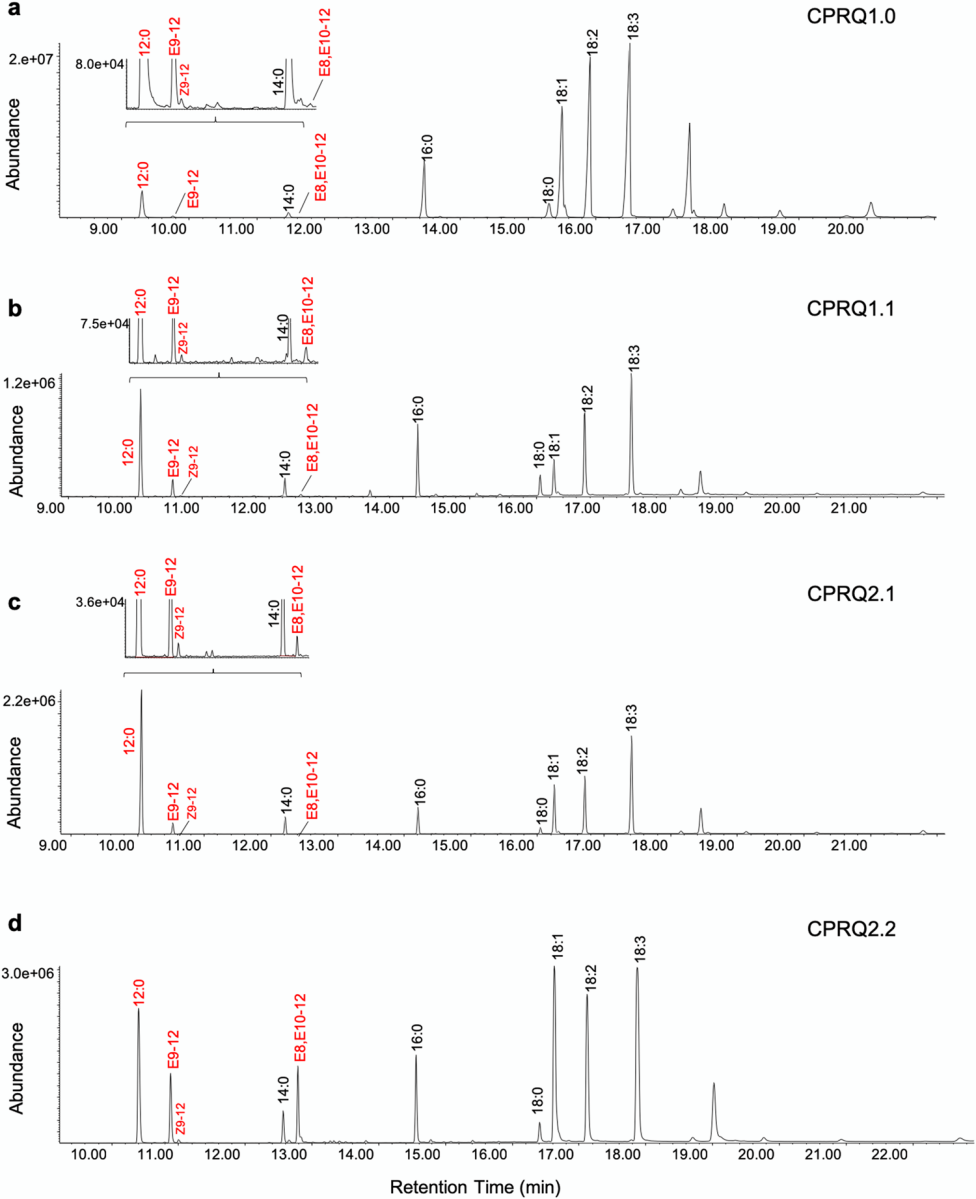
Chromatograms of fatty acid profiles in Camelina seeds from different transformants of **a** CPRQ1.0 (*UcTE—Cpo_CPRQ—AtWRINKLED1—CvLPAAT*); **b** CPRQ1.1 (*UcTE—Cpo_CPRQ*); **c** CPRQ2.1 (*UcTE—Cpo_CPRQ—UcTE—P19—Cpo_CPRQ*); **d** CPRQ2.2 (*UcTE—Cpo_CPRQ—Cpo_CPRQ—P19—Cpo_CPRQ*). The underlined *UcTE* indicates the transgene being present in the high lauric acid type Camelina seeds before transformation in this study. GC/MS analysis of total fatty acids in the form of corresponding methyl esters. 12:0, lauric acid; E9-12:acid, (*E*)-9-dodecenoic acid; and Z9-12:acid, (*Z*)-9-dodecenoic acid; E8,E10-12:acid, (*E*,*E*)-8,10-dodecadienoic acid; 14:0. myristic acid; 16:0, palmitic acid; 18:0, stearic acid; 18:1, oleic acid; 18:2, linoleic acid; 18:3, linolenic acid

**Fig. 5:**
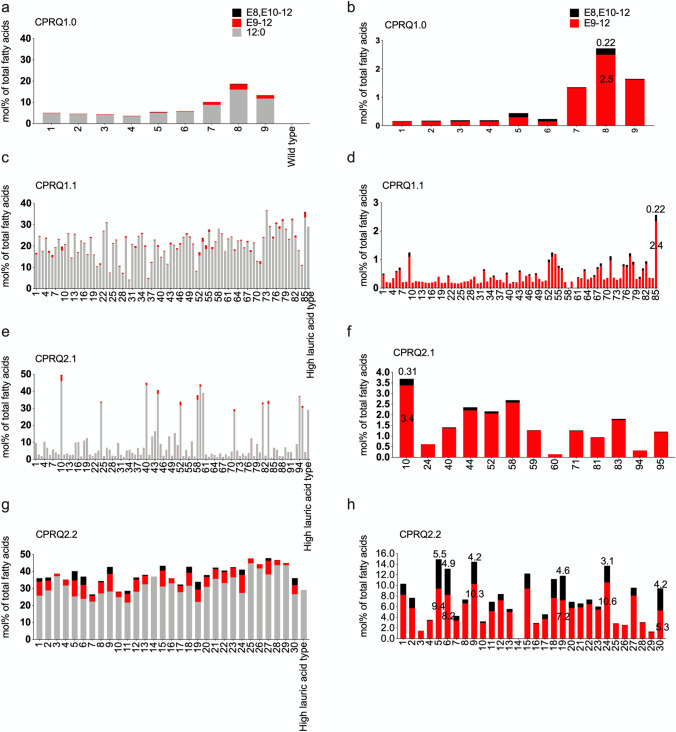
Percentage (mol%) of saturated and unsaturated C_12_ pheromone precursors of total fatty acids in pooled seeds of *C. sativa* in T_2_ generation from **a**, **b** CPRQ1.0 (*UcTE—Cpo_CPRQ—AtWRINKLED1—CvLPAAT*); **c**, **d** CPRQ1.1 (*UcTE—Cpo_CPRQ*); **e**, **f** CPRQ2.1 (*UcTE—Cpo_CPRQ—UcTE—P19—Cpo_CPRQ*); **g**, **h** CPRQ2.2 (*UcTE—Cpo_CPRQ—Cpo_CPRQ—P19—Cpo_CPRQ*)*.* The left column of figures include 12:0 and columns on the right do not. Fatty acids were analyzed in the form of corresponding methyl esters. 12:0, lauric acid; E9-12, (*E*)-9-dodecenoic acid; E8,E10-12, (*E*,*E*)-8,10-dodecadienoic acid. The underlined *UcTE* indicates the transgene being present in the high lauric acid type Camelina seeds before transformation in this study

**Fig. 6:**
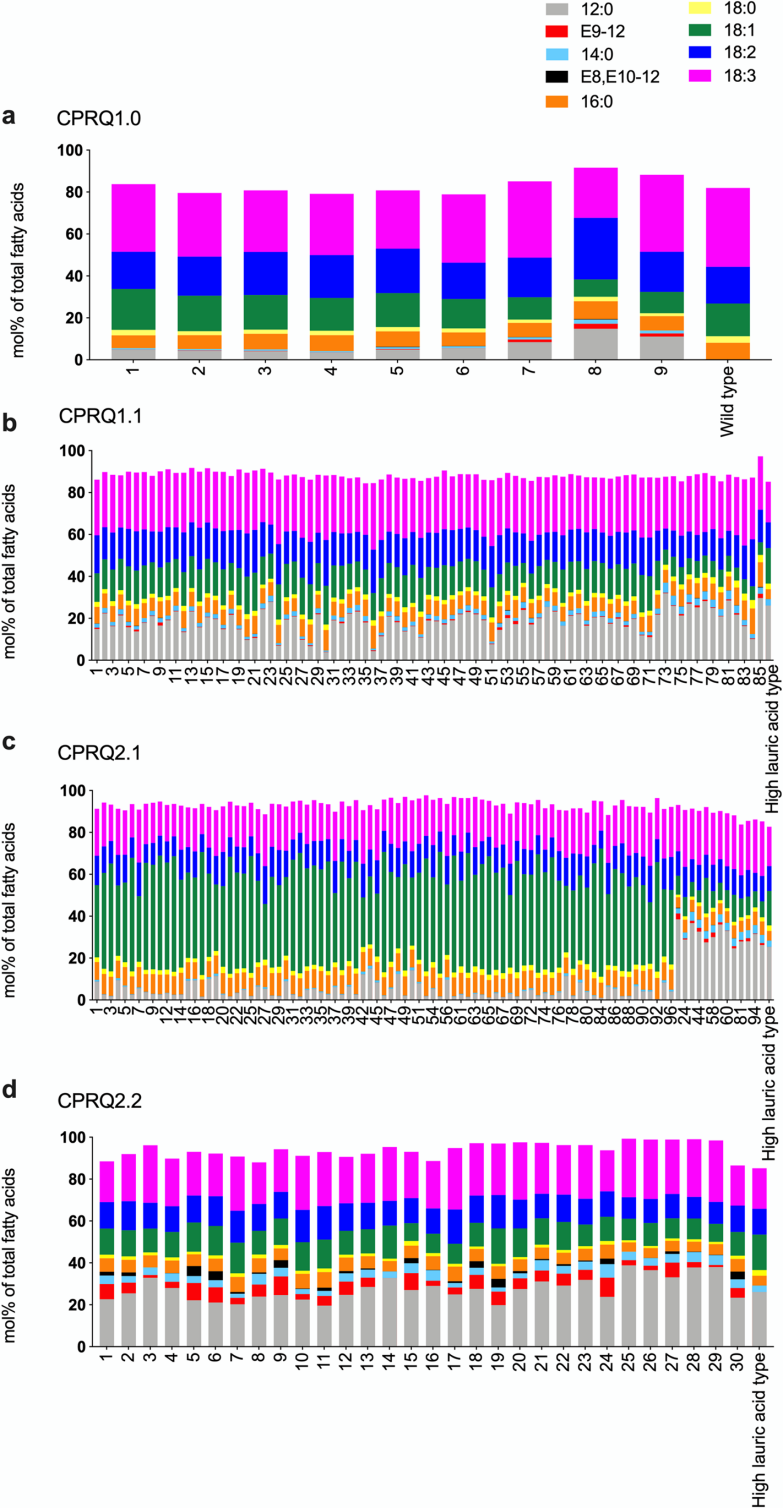
Percentage (mol%) of carbon length of C_12_ to C_18_ fatty acids of total methylated fatty acids in 25 pooled seeds from each transformant of *C. sativa* from **a** CPRQ1.0 (*UcTE—Cpo_CPRQ—AtWRINKLED1—CvLPAAT*); **b** CPRQ1.1 (*UcTE—Cpo_CPRQ*); **c** CPRQ2.1 (*UcTE—Cpo_CPRQ—UcTE—P19—Cpo_CPRQ*); **d** CPRQ2.2 (*UcTE—Cpo_CPRQ—Cpo_CPRQ—P19—Cpo_CPRQ*). The underlined *UcTE* indicates the transgene being present in the high lauric acid type Camelina seeds before transformation in this study. Fatty acids were analyzed in form of corresponding methyl esters. 12:0, lauric acid; E9-12, (*E*)-9-dodecenoic acid; 14:0. myristic acid; E8,E10-12, (*E*,*E*)-8,10-dodecadienoic acid; 16:0, palmitic acid; 18:0, stearic acid; 18:1, oleic acid; 18:2, linoleic acid; 18:3, linolenic acid

Among the engineered plants (Fig. 4) the fourth strategy produced the highest level of the C_12_ pheromone precursors (Fig. 4d). By analyzing 25 pooled seeds from each transformant, we found that among the nine transformants produced by introduction of gene cassette CPRQ1.0, the top-performing Camelina yielded 2.5% of E9-12:acid and 0.22% of E8,E10-12:acid from the total fatty acids (Fig. 5a, b). (Note: The percentage of fatty acids here and throughout this report was calculated as mole percent of total fatty acids). CPRQ1.1 was transformed into high lauric acid Camelina (Kim et al. 2015a), but the high concentration of 12:0 did not lead to higher production of the unsaturated products. In total, 85 transformants were obtained from CPRQ1.1, of which the best line produced 2.4% of E9-12:acid and 0.22% of E8,E10-12:acid (Fig. 5c, d). Similar to CPRQ1.1, the vectors of CPRQ2.1 and CPRQ2.2 were separately transformed into high lauric acid Camelina. In total, we obtained 96 and 30 transformants of CPRQ2.1 and CPRQ2.2, respectively. The analyses of the entire fatty acid profile (Fig. 6) showed that 83 of the CPRQ2.1 transformants (Fig. 6c) had reduced content of 12:0. It dropped from 29.0 to 4.5% and no unsaturated pheromone precursor was produced. The other 13 transformants (Fig. 6c) of CPRQ2.1 produced a higher amount of 12:0 and produced up to 3.4% of E9-12:acid and 0.31% of E8,E10-12:acid (Fig. 5e, f). In the CPRQ2.2 transformants, abundance of E9-12:acid and E8,E10-12:acid in 25 pooled seeds was 9.4% and 5.5%, respectively from the most productive plant (Fig. 5g, h).

### Comparison of Pheromone Precursors Production Between Four Strategies

**Fig. 7:**
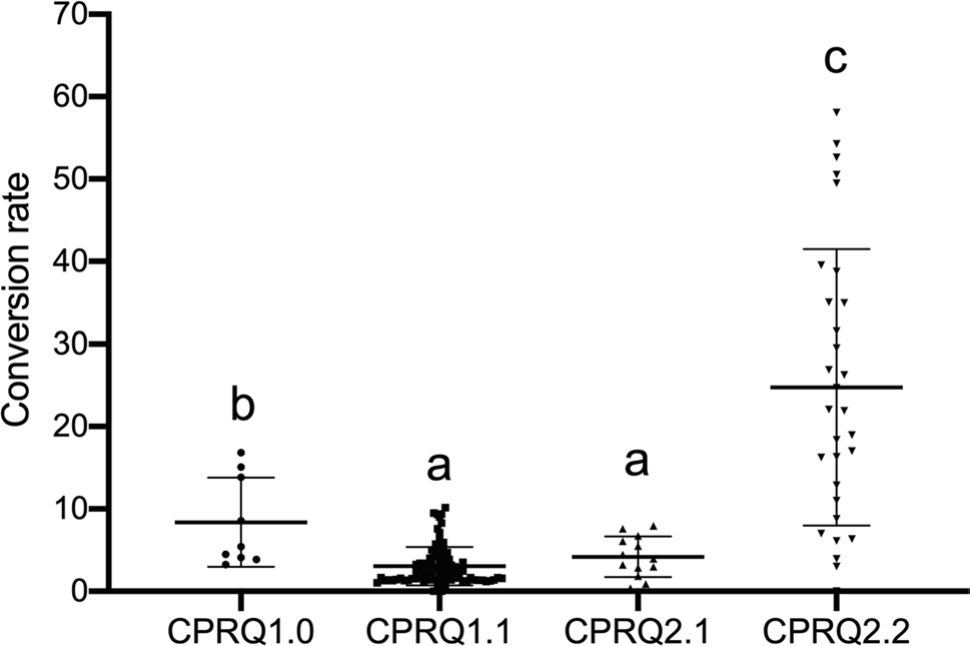
Conversion rate of lauric acid (12:0) into E9-12:acid and E8,E10-12:acid in four different types of T_2_ transformants. CPRQ1.0, *UcTE—Cpo_CPRQ—AtWRINKLED1—CvLPAAT*; CPRQ1.1, *UcTE—Cpo_CPRQ*; CPRQ2.1, *UcTE—Cpo_CPRQ—UcTE—P19—Cpo_CPRQ*; CPRQ2.2, *UcTE*—*Cpo_CPRQ—Cpo_CPRQ—P19—Cpo_CPRQ*. The underlined *UcTE* indicates the transgene being present in the high lauric acid type Camelina seeds before transformation in this study. Unpaired multiple t-test was used to compare the conversion rate between four types of transformants. The same letter above transformants indicates that the means are not significantly different (*P* < 0.05)

Comparing the amount of 12:0 and C_12_ pheromone precursors in CPRQ1.0 transformants to CPRQ1.1 and CPRQ2.1 showed that in CPRQ1.0 a higher percentage of 12:0 was converted into C_12_ unsaturated pheromone precursors (Fig. 7). The CPRQ2.2 transformants converted the highest percentage of 12:0 into corresponding pheromone precursors (Fig. 7).

**Fig. 8:**
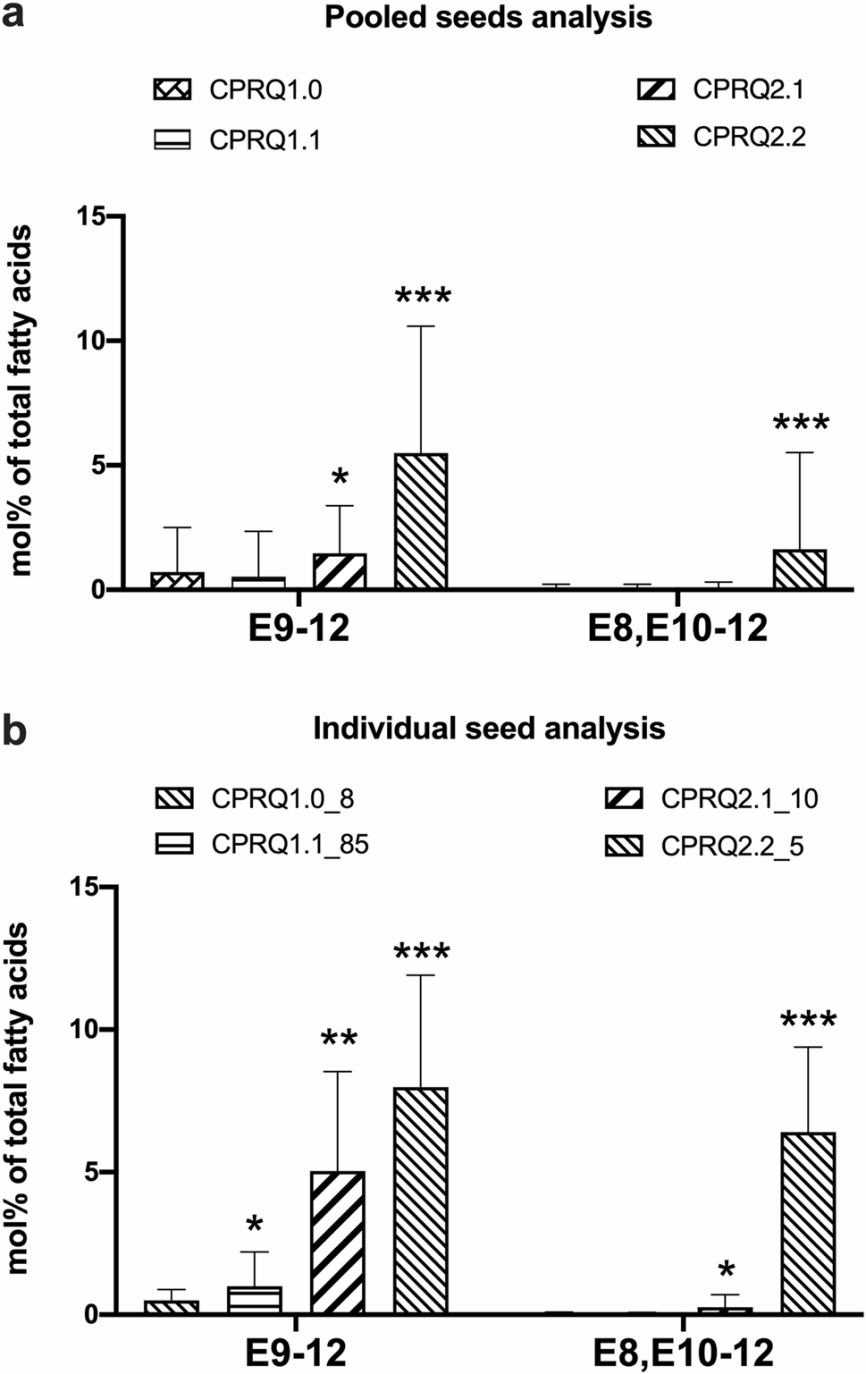
Comparison of mean percentage (mol%) of E9-12:acid and E8,E10-12:acid of total fatty acids between four strategies in T_2_ transformants. **a** Analyzed from pooled 25 seeds of each transformant; **b** analyzed from 15 individual seeds from most productive plant of each strategy. CPRQ1.0, *UcTE—Cpo_CPRQ—AtWRINKLED1—CvLPAAT*; CPRQ1.1, *UcTE—Cpo_CPRQ*; CPRQ2.1, *UcTE*—*Cpo_CPRQ—UcTE—P19—Cpo_CPRQ*; CPRQ2.2, *UcTE*—*Cpo_CPRQ—Cpo_CPRQ—P19—Cpo_CPRQ*. The underlined *UcTE* indicates the transgene being present in the high lauric acid type Camelina seeds before transformation in this study. Fatty acids were analyzed in form of corresponding methyl esters. Error bars indicate the mean ± range. Unpaired t-test was used to compare the production between four strategies. *, **, ***Indicate *P* < 0.01, *P* < 0.001, *P* < 0.0001

Compared to CPRQ1.0 and 1.1, the CPRQ2.1 and 2.2 transformants which contained multiple gene copies and *P19* increased the production of pheromone precursors. The most productive Camelina seed harboring CPRQ2.1 contained up to 8.5% of E9-12:acid and 0.7% of E8,E10-12:acid, which is higher than the best ones in CPRQ1.0 and CPRQ1.1 (Figs. 5 and 8). The Camelina expressing gene cassette of CPRQ2.2, which contained three copies of the ∆9 desaturase *CpCPRQ,* produced much higher amounts of E9-12:acid and E8,E10-12:acid than CPRQ2.1 that had two copies of *CpCPRQ* (Figs. 5 and 8). In addition, no abnormal plant development was observed among CPRQ2.1 and CPRQ2.2 transformants.

### Increase of Oleic Acid in CPRQ2.1 Transformants

As mentioned above, the CPRQ2.1 transformants showed two different fatty acid profiles (Fig. 6c). In the first profile group, the 12:0 or 18:3 was the dominant fatty acid, followed by 18:2 and 18:1, which is similar to other transformants (CPRQ1.0, CPRQ1.1, CPRQ2.2), the wild type and the high lauric type (Fig. 6a, b, d). In the second profile group (13 of the CPRQ2.1 transformants), oleic acid (18:1) was the dominant fatty acid species and there was a decreased amount of 12:0. Moreover, the ones that lost the 12:0 did not produce any unsaturated C_12_ pheromone precursors.

### C_12_ Pheromone Precursors were Stably Produced in T_4_ Generation Plants

**Fig. 9:**
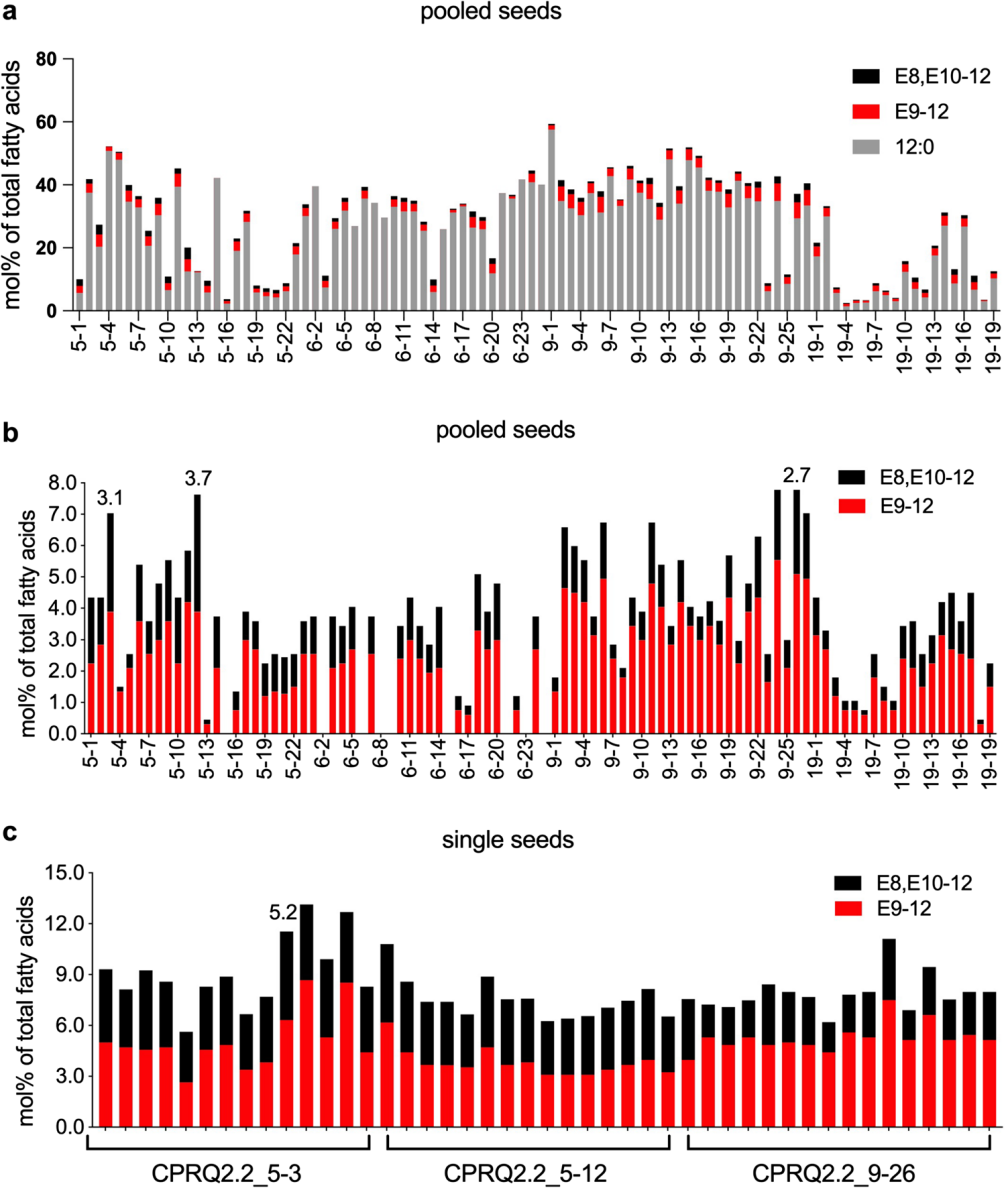
Percentage (mol%) of saturated and unsaturated C_12_ pheromone precursors of total fatty acid in T_3_ seeds from CPRQ2.2 (*UcTE*—*Cpo_CPRQ—Cpo_CPRQ—P19—Cpo_CPRQ*). **a** and **b**, 25 pooled seeds; **c** individual seeds*.* The underlined *UcTE* indicates the transgene being present in the high lauric acid type Camelina seeds before transformation in this study. Fatty acids were analyzed in the form of corresponding methyl esters. 12:0, lauric acid; E9-12, (*E*)-9-dodecenoic acid; E8,E10-12, (*E*,*E*)-8,10-dodecadienoic acid

**Fig. 10:**
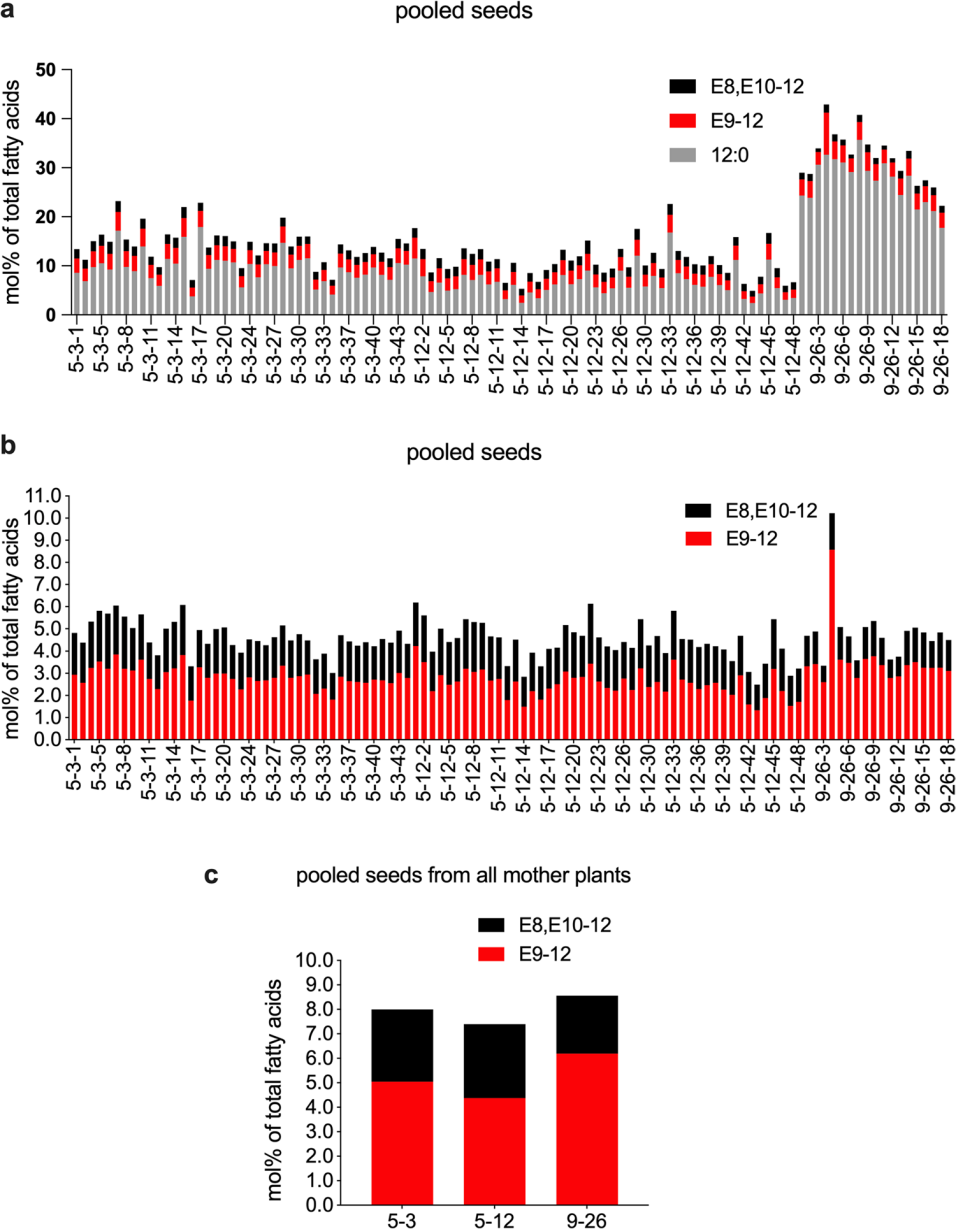
Percentage (mol%) of saturated and unsaturated C_12_ pheromone precursors of total fatty acid in T_4_ seeds from CPRQ2.2 (*UcTE*—*Cpo_CPRQ—Cpo_CPRQ—P19—Cpo_CPRQ*)*.* **a** and **b**, pooled seeds from each plant; **c** pooled seeds from all plants which are the offspring of the same T_3_ plant. The underlined *UcTE* indicates the transgene being present in the high lauric acid type Camelina seeds before transformation in this study. Fatty acids were analyzed in the form of corresponding methyl esters. 12:0, lauric acid; E9-12, (*E*)-9-dodecenoic acid; E8,E10-12, (*E*,*E*)-8,10-dodecadienoic acid

The production of the unsaturated pheromone precursor E9-12:acid and E8,E10-12:acid in CPRQ2.2 T_2_ seeds ranged from 1.2% to 10.6% and 0.01% to 5.51%, respectively, of total fatty acids (Fig. 5h). In CPRQ2.2 T_3_ seeds, the variability was reduced but still high. The production of E8,E10-12:acid ranged from 0.15 to 3.74% of total fatty acids (Fig. 9). In CPRQ2.2 T_4_ seeds after three generations of selfing, we found that the variability of the production of C_12_ pheromone precursors from the same mother plant (T_3_ plant) was small (Fig. 10). The di-unsaturated pheromone precursor E8,E10-12:acid was stably produced at levels from 2 to 3% (Fig. 10).

The average level of the target acid in T_4_ seeds from lines CPRQ2.2_5-3, CPQR2.2_5-12 and CPRQ2.2_9-26 were 2.6%, 2.6% and 1.8%, respectively (Fig. 10b). It is worth mentioning that all the T_4_ seeds of selected CPRQ2.2 lines 5–3, 5–12, 9–26 show a phenotype of red colour, except for seeds from two individual plants numbered 5–3–21 and 5–3–36, which have normal light brown colour. Further GC/MS analysis showed that only the seeds from these two plants did not produce any lauric acid, monounsaturated and doubly unsaturated C_12_ acid.

### Conversion of Fatty Acids into Corresponding Alcohols

**Fig. 11:**
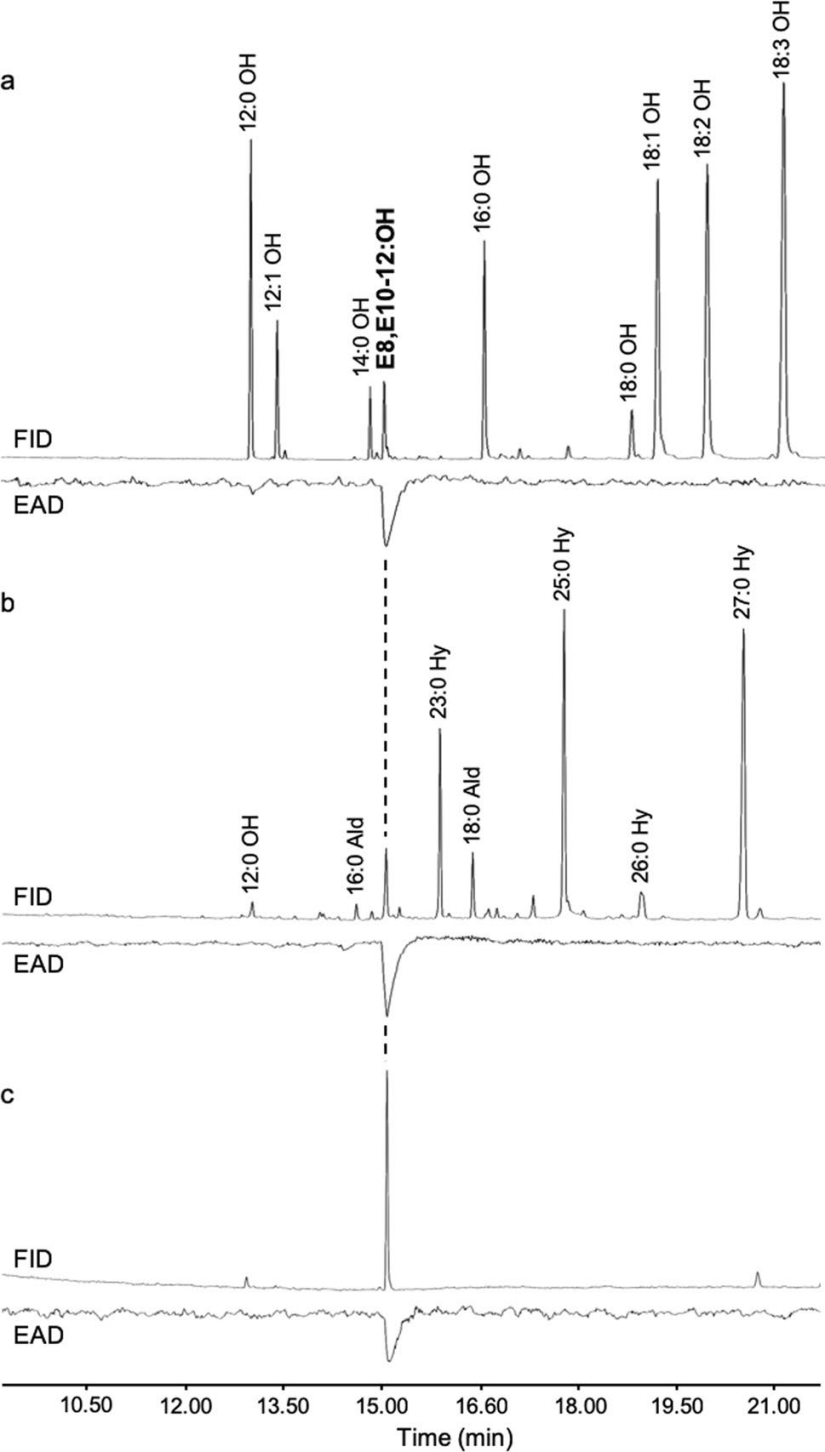
Gas chromatographic analysis of plant and insect derived pheromone and synthetic pheromone simultaneously using a flame ionization detector (FID) and a male *C. pomonella* antennae as an electroantennographic detector (EAD). **a** Alcohol products converted from seed oil fatty acids of a stably transformed *C. sativa* line expressing a thioesterase and a desaturase (Cpo_CPRQ) elicit an antennal response at the retention time corresponding to the pheromone component (*E*,*E*)-8,10-dodecadienol (E8,E10-12:OH); **b** antennal response to the female pheromone gland extract (1/5 female equivalent); **c** antennal response to the synthetic codlemone

Methyl esters of the fatty acids including the target diene precursor were reduced into corresponding alcohols. The plant-derived sample contained large amounts of saturated alcohols of different chain length as well as the oleyl, linolyl and linolenoyl alcohols, in addition to the E9-12:OH and the codlemone (Fig. 11). The relative abundance of plant-derived codlemone was 1.5% wt, similar to the abundance of the diene acid (1.7% wt) in the fatty acids from the seeds. The isomeric purity of 8,10-dodecadienol isomers was 81.2% EE, 7.4%EZ, 9.9%ZE, and 1.5%ZZ. After AgNO_3_-silica column fractionation, the target alcohol was isolated with a chemical purity of 85.6% and isomeric purity of 94.3% EE, 2.3% EZ, 3.1% ZE, and 0.3% ZZ.

### GC-EAD Analyses, Flight Tunnel Assay and Field Trapping

GC-EAD analyses showed strong antennal responses to codlemone both in the plant-derived alcohols and in female gland extract, as well as to the synthetic compound (Fig. 11). Notably, the antennae did not respond to the other abundant components in the plant-derived alcohol sample with the exception of the saturated dodecanol. This compound was approximately four times as abundant as our target compound, but it still evoked only a minor antennal response.

**Fig. 12:**
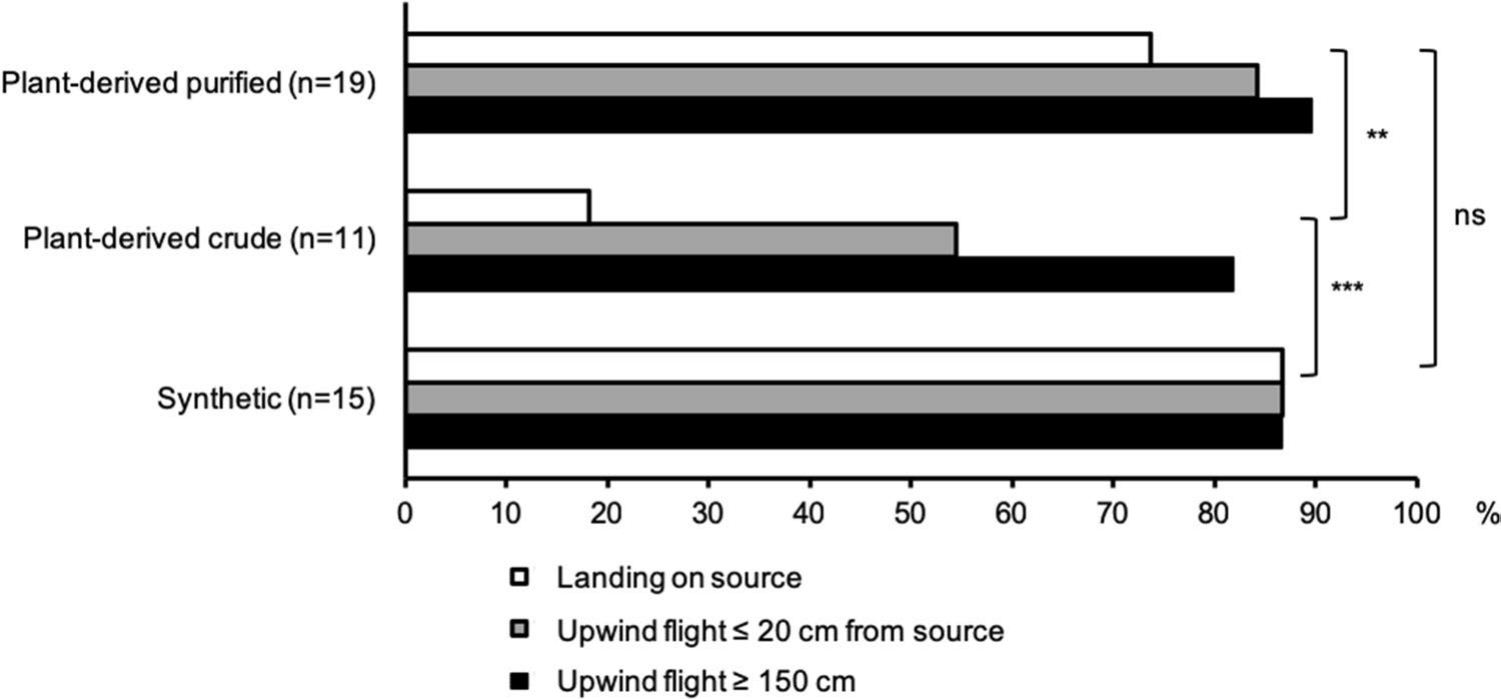
Flight tunnel assay of male codling moths *C. pomonella* responding to codlemone of different quality. Rubber septa loaded with 100 μg of the active ingredient, E8,E10-12:OH (synthetic, plant-derived crude or purified plant-derived alcohols) were tested. Bars indicate the percentage of males flying upwind 150 cm (black), upwind flight to 20 cm or less from the source (grey), and landing at the source (white). Asterisks indicate significant differences in landing at the source, according to a Chi-square analysis (**P < 0.01, ***P < 0.001)

In the flight tunnel assay, upwind flight in the odor plume was observed for > 80% of the male moths regardless of stimulus (Fig. 12). There was no significant difference in source contact rate between males tested against synthetic codlemone and plant-derived and purified codlemone (χ^2^ = 0.86; d.f. = 1; *P* = 0.353). In contrast, the source contact rate of males tested against the plant-derived crude codlemone was significantly lower than for both synthetic codlemone (χ^2^ = 12.19; d.f. = 1; *P* < 0.001) and plant-derived and purified codlemone (χ^2^ = 8.62; d.f. = 1; *P* < 0.01).

**Fig. 13:**
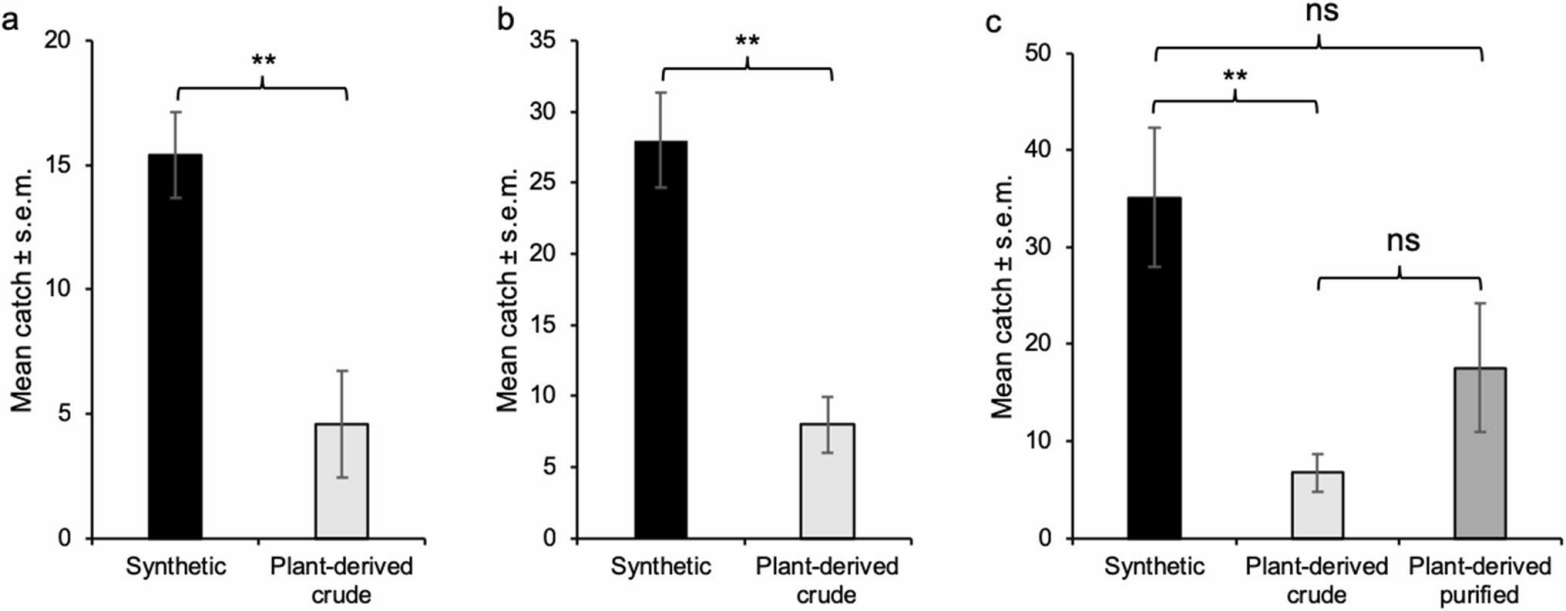
Field trapping of codling moth *C. pomonella*. Males caught in traps baited with synthetic or plant-derived crude codlemone (100 µg of active ingredient per lure) in an apple orchard (**a**) (N = 5), and in home gardens in Lund municipality (**b**) (N = 4) in Sweden, June 9th to 17th, 2021. Catches were analyzed using t-test on log(x + 1)-transformed data (**P < 0.01). Males caught in traps baited with synthetic, plant-derived crude or plant-derived and purified codlemone (100 µg of compound per lure) in home gardens in Lund municipality (**c**) (N = 7), Sweden, June 24th to 28th, 2021. Catches were analyzed using Anova followed by Bonferroni *post-hoc* test on log(x + 1)-transformed data (**P < 0.01)

In a first round of field trapping experiments, attraction to the crude plant-derived codlemone and a sample of synthetic codlemone was compared. Both treatments attracted *C. pomonella* males specifically. No males were caught in the unbaited control traps in any of these experiments, and this treatment was excluded from the statistical analysis. In the first orchard (N = 5), there was a significantly higher attraction of males to synthetic codlemone vs. plant-derived codlemone (t = 3.88; d.f. = 8; *P* < 0.01) (Fig. 13a). In the second orchard (N = 5), the catches were too low for any meaningful analysis of statistical significance. Traps baited with synthetic pheromone trapped in total 13 males whereas plant-derived codlemone attracted 7 males. Finally, in the home garden experiment (N = 4), significantly higher attraction of males was observed to synthetic codlemone vs. plant-derived codlemone (t = 3.80; d.f. = 6; *P* < 0.01) (Fig. 13b). In the second round we investigated the impact of further purification on the attractivity of the plant-derived pheromone (N = 7). There was no significant difference in attraction of males to synthetic vs. purified plant-derived codlemone (*P* = 0.20) or between the two plant-derived stimuli (*P* = 0.70) (Fig. 13c), whereas synthetic codlemone attracted more males than the crude plant-derived codlemone (*P* < 0.01).

## Discussion

The codling moth is one of the major targets for pheromone-based IPM control strategies and the major target for mating disruption with pheromones in orchards (Witzgall et al. 2008). The first effective pheromone dispenser for mating disruption of codling moth was registered in the United States in 1991 and since then mating disruption with synthetic codlemone has become an integral part of codling moth management in apple and pear orchards in many regions. Reviewing the impact of sex pheromones in pest control, Witzgall et al. (2010) reported that 210,000 ha of orchards were treated worldwide with mating disruption against codling moth, an area second only to the areas treated against the gypsy moth *Lymantria dispar*. The annual codlemone production in 2006 was estimated to 25 metric tons (ibid, based on data provided by Shin-Etsu Chemical Co., Tokyo, the major producer of synthetic codlemone) and is now likely much higher. We have demonstrated a green chemistry alternative to conventional pheromone synthesis using a metabolically engineered oilseed plant factory to provide the immediate fatty acid precursor of codlemone.

The production of mono- and di-unsaturated C_12_ moth pheromone precursors in Camelina seeds confirmed the “*in-planta*” function of the ∆9 desaturase derived from *C. pomonella.* When *Cpo_CPRQ* was expressed in Camelina it could produce E9-12:acyl from lauric acid as well as a small amount of the corresponding *Z* isomer. Furthermore, it had the activity to convert the monoene intermediate into E8,E10-12:acyl with conjugated double bonds. This gene bifunctionality is consistent with the previous study of Lassance et al. (2021), using the sf9 insect cell expression system. Our results substantiate the usefulness of the plant expression system for functional characterization of insect pheromone biosynthetic genes (Löfstedt and Xia 2021), and furthermore demonstrate the feasibility of production of diene moth sex pheromone precursors in a plant. We demonstrated that co-expression of the desaturase with *P19* and multiple gene copies can increase the production of C_12_ pheromone precursors significantly. Also, it was confirmed that stably expressing *P19* regulated by the seed-specific *napin* promoter would not cause observable harm of plant development.

We established four types of transformant lines by using different exogenous gene cassettes. In the engineered Camelina seeds, the mono-unsaturated E9-12:acid with small amount of (*Z*)-9-dodecenoic acid (Z9-12:acid) and diunsaturated E8,E10-12:acid were produced in all four types of transformant lines (Fig. 4). Comparing the fatty acid profiles of transformants from CPRQ1.0 and CPRQ1.1 suggests that co-expression with the transcription factor WRINKLED1 from *Arabidopsis AtWRI1* and one of the Kennedy pathway genes lysophosphatidic acid acyltransferase from *C. viscosissima CvLPAAT* (Kim et al. 2015b) can improve the substrate utilization of 12:0. For example, the mean conversion ratio of 12:0 into unsaturated pheromone precursors in CPRQ1.0 was 7.5%, while in CPRQ1.1 transformant it was 2.9%. Also, this implied that the bottleneck of production of pheromone precursors was the activity of *Cpo_CPRQ*, rather than the amount of 12:0 substrate available. Among 85 transformants from CPRQ1.1, containing ca. 23% of 12:0, the most productive plant produced even lower amounts of E9-12:acid and same level of E8,E10-12:acid as compared to CPRQ1.0, containing at the most 16.0% of 12:0 in nine transformants.

The Cpo_CPRQ desaturase showed high activity towards production of E9-12:acid and E8,E10-12:acid in insect cells (Lassance et al. 2021). So why did *Cpo_CPRQ* show a lower activity in Camelina CPRQ1.0 and 1.1 transformants? One explanation could be that the potential silencing effect on repeated transgenes in Camelina may cause a shut-down of the exogenous expression (Hagan et al. 2003; Schubert et al. 2004). By expressing two other gene cassettes (CPRQ2.1 and CPRQ2.2) in Camelina seeds, we concluded that the low activity of *Cpo_CPRQ* might indeed be caused by transgene silencing. Both CPRQ2.1 and CPRQ2.2 contained the gene encoding P19, a viral silencing suppressor protein (VSP) from tomato bushy stunt virus (TBSV), which was reported to have the ability to suppress the silencing effect of transgenes in previous studies. Naim et al. (2016) demonstrated that expression of the silencing-suppressor protein can protect and enhance stable transgene performance. The expression of CPRQ2.1 and CPRQ2.2, both with P19, did increase the production of E/Z9-12:acid and E8,E10-12:acid significantly (Fig. 7).

The VSPs, including P19 (Wood et al. 2009), V2 (Wartig et al. 1997), P0^PE^ (Fusaro et al. 2012), have been widely used together with transgene cassettes to enhance their expression during transient expression experiments. However, for stable transformation, the VSPs are rarely used and explored as they may interfere with endogenous microRNA-regulated processes and lead to abnormal plant development (Dunoyer et al. 2004; Fusaro et al. 2012). In the present study, we investigated the use of fully functional *P19* expressed only in seeds and controlled by the *napin* promoter. We demonstrated that stably expressed *P19* in Camelina seeds with *napin* promoter did not cause any abnormal plant development. The germination rate of the most productive seeds from CPRQ2.2 was as high as 95%. Thus, using the *napin* promoter to control the expression of P19 would seemingly not disrupt the small RNA driven regulatory pathways towards the development of vegetative shoot in Camelina (Wong et al. 2011).This may be due to the native *napin* promoter driving the seed storage proteins like napin, whose physiological role is to provide the growing seedling with essential nutrients prior to the establishment of the photosynthetic capacity (Rask et al. 1998).

We were surprised to find that the best individual seed from the CPRQ2.1_10 transformant only produced 0.7% of E8,E10-12:acid, while the best seed from CPRQ2.2_5 transformant produced 8.5% of this compound. This demonstrates that multiple gene copies perform well in Camelina towards the di-unsaturated pheromone precursor production. The ten times higher production amount in CPRQ2.2 might benefit from the additional copy of *Cpo_CPRQ* but influence of the insertion site of the transgene in the Camelina genome cannot be ruled out (Fig. 7). By cultivating the subsequent generations to homozygosity, we also confirmed that the C_12_ pheromone precursors in seeds can be stably produced although the amount of the precursors showed big variation between individual seeds and average titre decreased somewhat in later generation, showing that there is still room for improvement. Interestingly, all the T_4_ seeds of selected CPRQ2.2 lines (5–3, 5–12, 9–26) show a phenotype of red colour except for the seeds from two individuals which correlated with loss of the production of lauric acid, as well as monounsaturated and doubly unsaturated C_12_ acid. The reason for this observable phenotypic trait is not clear but it might still be used as a quick selection marker for presence/absence of C_12_ pheromone precursors in seeds in the future breeding of the CPRQ lines.

When compared to synthetic codlemone, the plant-derived codlemone performed well without purification, both in the flight tunnel assay and in the field experiments considering that the sample contained very large amounts of saturated alcohols of different chain-length as well as the oleyl, linolyl and linolenoyl alcohols, in addition to the E9-12:OH and the codlemone. The overall purity of codlemone (E8,E10-12:OH) in this unprocessed batch was only 2.5% and the isomeric purity (%EE isomer) was 81%. Still this bait trapped 30% as many males as the conventionally produced codlemone which had an overall chemical purity above 95% and isomeric purity of 98%. The purified plant-derived codlemone obtained after column chromatography performed even better and trapped 50% as many males as the conventionally produced codlemone. Column chromatography increased the overall purity of the plant-derived codlemone to ca 86% and removed virtually all the long-chain alcohols. The isomeric purity remained lower (94%). The EAD analysis did not reveal any EAD active impurities in addition to 12:OH and the presence of 12:OH may not even be a problem. Dodecanol is present also in pheromone gland extracts (Fig. 11), albeit in lower relative amounts, and addition of this compound actually increased the attractiveness of the codlemone (Arn et al. 1985).

Codlemone as well as other doubly unsaturated pheromone components with conjugated double bonds is prone to isomerization. The equilibrium mixture of isomers of 8,10-dodecadienol is 61% EE, 20% EZ, 14% ZE and 5% ZZ according to McDonough et al. (1996). Female codling moths produce minor amounts of the geometric isomers of codlemone (Arn et al. 1985) and codlemone isomerizes on the surface of dispensers used for trapping and mating disruption (Brown et al. 1992). Small amounts of the isomers do not decrease attraction to codlemone lures but the equilibrium isomer blend is a much weaker male attractant. In the field, attraction to equilibrium baits was less than 1/3 of the attraction to pure codlemone (El-Sayed et al. 1998). It is thus likely that the lower attractiveness of the plant-derived codlemone to a large extent may be explained by its lower isomeric purity. When it comes to using codlemone for mating disruption isomeric purity is, however, not a concern. McDonough et al. (1996) reported that percentage disruption was even higher with the equilibrium mixture than with the pure EE isomer.

In conclusion, we demonstrated the feasibility of producing mono- and di-unsaturated C_12_ moth pheromone precursors in transgenic plants. Using Camelina as the production platform for E8,E10-12:acid, we obtained levels of ≥ 8.5% of total fatty acids in seeds of the T_2_ generation. Even though the production amount of these C_12_ pheromone precursors decreased somewhat in the following generations, a stable pheromone-producing Camelina line was still obtained. Because the oil content of the Camelina seeds is typically between 35 and 45% of the dry wt and the yields of Camelina range from 336 to 2240 kg of seeds per hectare (Moser 2010), this translates into the potential production of 7.4 to 63.5 kg (minimum to maximum) of E8,E10-12:acid by cultivation of our best Camelina line, which is a promising starting point for optimization and potential commercialization of a plant factory for codlemone production.
